# Lead Water-Pipes

**Published:** 1871-06

**Authors:** 


### LEAD WATER-PIPES.

The public are hereby warned against using lead water-pipes lined with tin or zinc, or any other metal; two metals touching each other, kept in water, will rust,—oxidize by galvanic action, and by this decomposition will become poisonous; and lead is only poisonous by being decomposed. Besides, all linings of tin are liable to crack; and wherever there is a crack, there is corrosion, and eventually a leak.

After a lead pipe has conveyed water for a great while, a coating forms, which in a measure becomes impervious to air.

Put muriatic acid on lead shavings, and they begin to dissolve, a snowy crust forms; but after a while the strongest acid will not corrode the lead, because there is a crust on it which no water can work away, which cannot be got rid of in any other way yet known than by melting the lead up. Hence the great noise made by many as to the poisonous effects of lead water-pipes in cities is without adequate foundation. In the largest cities people live the longest, and large cities have their water come to their houses in lead pipes.

Here is a broad fact: the city of Aberdeen, Scotland, uses a million gallons of water every day, distributed to dwellings by lead pipes. Chemical analysis shows that there is as much as thirty-three one hundredths of a grain of lead in each gallon, and yet in fifteen years not one case of lead poisoning has come to the knowledge of the authorities. This need not conflict with the idea above that the lead does not decompose, because it does decompose for a while; besides, old pipes are giving out all the time, and new houses are being built requiring new lead pipes. Until glass pipes can be used, perhaps lead pipes are best; at all events, do not use lead pipes lined with any other metal.

